# Cyclohexyl acetate functions like a volatile sex pheromone mimic in *Caenorhabditis* nematodes

**DOI:** 10.1186/s12915-026-02510-0

**Published:** 2026-01-15

**Authors:** Xuan Wan, Yuki Togawa, Matthew R. Gronquist, Marika Sagawa, Daniel Leighton, Chung Man Chan, Frank C. Schroeder, King L. Chow, Paul W. Sternberg, Ryoji Shinya

**Affiliations:** 1 Division of Biology and Biological Engineering, California Institute of Technology, Pasadena, USA; 2 School of Agriculture, Meiji University, Chiyoda, Japan; 3 Department of Chemistry and Biochemistry, State University of New York at Fredonia, Fredonia, USA; 4 Present address: Department of Human Genetics, Department of Biological Chemistry, and Howard Hughes Medical Institute, University of California, Los Angeles, USA; 5 Division of Life Science and Department of Chemistry & Biology Engineering, Hong Kong University of Science and Technology, Hong Kong, China; 6 Boyce Thompson Institute and Department of Chemistry and Chemical Biology, Cornell University, Ithaca, USA

**Keywords:** *Caenorhabditis* nematodes, Volatile sex pheromone, Mate searching, Pheromone mimic, Sensory neurons

## Abstract

**Background:**

Nematodes communicate via diverse sex pheromones, including long-range volatile signals, short-range chemical cues, and contact-dependent molecules. While the ascaroside family of small molecules that mediate short-range attraction is well characterized, the identities and roles of volatile sex pheromones (VSPs) that act over longer ranges remain unknown.

**Results:**

Using GC–MS analysis of crude VSP extracts, we identified cyclohexyl acetate (CA) as a candidate mimic, sharing retention time and mass spectral features with natural VSPs. Behavioral assays demonstrated that CA acts as a concentration-dependent, male-specific attractant in *Caenorhabditis.* Pre-exposure to VSPs induced cross-adaptation to CA, suggesting shared sensory processing. Surprisingly, genetic and calcium imaging analyses revealed that CA perception is mediated primarily by AWC_on_ (*str-2*-expressing) neurons and involves VSP chemoreceptor *srd-1*-independent pathways, which are distinct from the neural pathways involved in natural VSP perception.

**Conclusions:**

These data indicate that CA is unlikely to be a major VSP constituent; rather, it is a structural analog that elicits male-specific attraction via a parallel sensory circuit. The endogenous source of CA in *C. remanei* remains unresolved; our data do not establish whether females produce CA. Its structural and behavioral mimicry provides new insights into the complexity of chemosensory signaling and the potential for interspecies chemical eavesdropping in nematode ecology.

**Supplementary Information:**

The online version contains supplementary material available at 10.1186/s12915-026-02510-0.

## Background

Communication via chemical cues is ubiquitous across the animal kingdom. Small molecule chemical signals mediate a variety of intraspecific and interspecific interactions, such as mate location and choice and predator–prey encounters. In the nematode *Caenorhabditis elegans*, a family of small molecules, the ascarosides, controls sexual attraction, development, and social behavior [1, 2]. Blends of these compounds, dauer pheromones, integrate environmental cues like population density and food availability to trigger larval entry into the stress-resistant dauer stage, a key survival adaptation under adverse conditions [3, 4]. Beyond developmental regulation, ascarosides influence behaviors ranging from mate attraction and aggregation to repulsion and exploratory movement [2, 5]. Some exhibit concentration-dependent function; for example, ascr#2 and ascr#3 attract males at low concentrations but repel hermaphrodites at higher concentrations [4, 5]. This functional versatility shows the complicated concentration-dependency of pheromone communication [6–11].

While nonvolatile pheromones such as ascarosides mediate contact-dependent, short-range communication, *C. elegans* also utilizes volatile sex pheromones (VSPs) for long-range mate attraction. VSPs can attract males from distances of at least 10 cm under laboratory conditions [12]. Although ascarosides also can diffuse through the environment, they are generally classified as nonvolatile and require direct contact or proximity to trigger behavioral responses [2, 5, 13]. Among ascarosides, only certain mate-searching compounds such as ascr#2, ascr#3, and ascr#4 are active over relatively longer ranges [2, 14].

The VSP facilitates mate attraction and provides critical information about an individual’s sex, reproductive status, and developmental stage [12]. The existence of long-range volatile signals is supported by observations that certain chemical communications persist even in mutants deficient in ascaroside production [15, 16]. Virgin females from the *Caenorhabditis* species *C. elegans*,* C. remanei*, and *C. brenneri* secrete VSPs that are attractive to males from the closely related androdioecious species *C. elegans and C. briggsae*, as well as the dioecious *C. brenneri* and *C. remanei*. The production of VSPs is both sex- and stage-specific. In *C. elegans* hermaphrodites, oocyte–somatic communication regulates the synthesis of mate-attracting VSPs. These volatiles are produced only by virgin *fog-2* mutant females or by hermaphrodites that have exhausted their self-sperm [12, 15].

Sexual attraction in *C. elegans* is mediated by a combination of sex-specific and core sensory neurons. Male-specific neurons, such as CEMs, and sex-shared sensory neurons, such as AWAs, contribute to robust male attraction [15, 17–19]. The male response to volatile cues relies on molecular pathways involving G protein-coupled receptors and TRPV channels, such as OSM-9 [15, 17, 18]. Moreover, the sexually dimorphic expression of receptors, such as SRD-1 only in the AWA neurons of males, enhances male sensitivity to VSP signals, reflecting the nuanced regulation of chemosensory signaling [18].

Chemical signaling is highly conserved in nematodes [20], and other species have evolved the ability to detect and respond to these signals [21, 22]. The influence of *C. elegans* pheromones extends beyond nematodes, affecting interkingdom interactions. For example, nematophagous fungi, which are natural predators of soil-dwelling nematodes, can detect and respond to nematode-secreted ascarosides to induce trap formation, a coevolved predator–prey relationship [23]. Similarly, plants, including *Arabidopsis*, tomato, potato, and barley, respond to ascarosides such as ascr#18 by activating robust defense mechanisms against a wide range of pathogens, including viruses, bacteria, and fungi [24]. Plant-parasitic nematodes utilize ascarosides to promote the reproductive development of their vector beetles, which, in turn, secrete ascarosides to attract nematode larvae for dispersal [25].

The lack of clarity regarding the identities of VSPs produced by *C. elegans* and related species represents a significant gap in our understanding of communication within nematodes. While over 200 ascaroside variants have been characterized [26], the identities of VSPs remain elusive despite close to two decades of research and the identification of numerous candidate compounds. To address this gap, we conducted a targeted screen of potential VSP analogs. Here, we report the identification of cyclohexyl acetate (CA) as a chemical mimic of the volatile, long-range, male-attractive pheromone found among *Caenorhabditis* nematodes.

## Results

### Identification of a female-specific volatile compound in *Caenorhabditis* via GC–MS

**Fig. 1:**
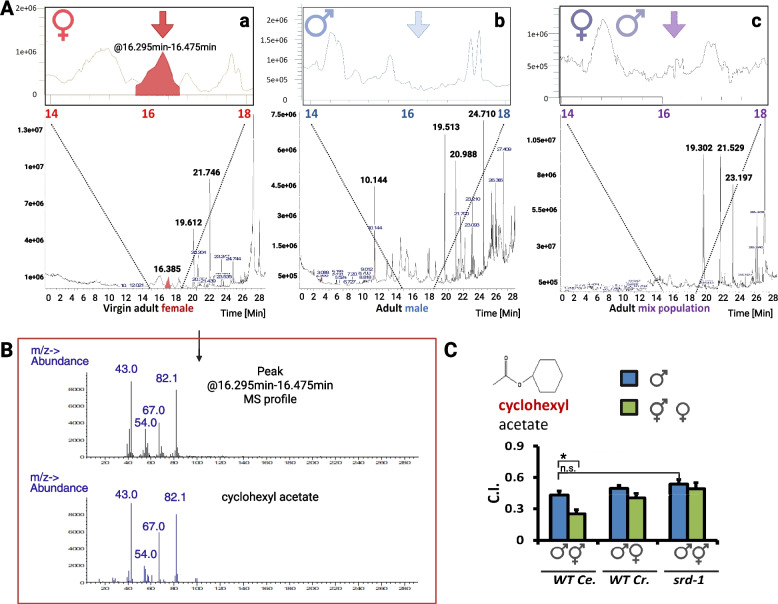
**A** Gas chromatography (GC) profiles of extracts from *C. remanei* (EM464) adult worms. (a) Virgin female extract showing a specific peak (red arrow) at retention time 16.295–16.475 min. (b) Male extract lacking the female-specific peak. (c) Mixed-population extract confirming the absence of the peak in the 16.295–16.475 min window. Mass spectrometry (MS) analysis of this region in (b) and (c) showed no match to the female-specific compound. **B** Mass spectrometry (MS) validation. Upper panel: MS profile of the female-specific peak (16.295–16.475 min). Lower panel: Reference MS profile of cyclohexyl acetate standard. All major peaks align. **C** Chemotaxis indices of wild-type *C. elegans* and *C. remanei* in response to 1000-fold diluted cyclohexyl acetate. Error bars denote standard error. * Indicates a significant difference (*P* < 0.05)

Gas chromatography (GC) of one-day-old adult *C. remanei* revealed a female-specific compound in virgin female extracts, evident as a distinct peak at 16.295–16.475 min on a DB-17 ms capillary column (30 m × 0.32 mm) and at 31.26 min on a DB-624 capillary column (30 m × 250 µm × 1.40 µm) (Fig. 1A, panel a; Additional file 1: Fig. S1). This peak was absent from extracts of males (Fig. 1A, panel b; Additional file 1: Fig. S1) and mixed populations (Fig. 1A, panel c; Additional file 1: Fig. S1), indicating sex- and reproductive-state specificity. Both GC–MS conditions are described in the “Methods” section. Mass spectrometry (MS) analysis of the corresponding GC region in male and mixed samples confirmed the absence of this compound. Virgin females of *Caenorhabditis* species (*C. elegans*,* C. remanei*,* C. briggsae*, and* C. brenneri*) secrete VSPs that attract conspecific and heterospecific males [15]. Prior studies revealed that VSP production in *C. remanei* exceeds that of *C. elegans* by at least 20-fold [12, 15, 27]. Given the GC–MS requirement for large-scale sample preparation (5,000 virgin females *C. remanei* to achieve detectable signal-to-noise ratios), *C. remanei* was selected for VSP extraction. Mass spectrometry profiling of this female-specific peak yielded a fragmentation pattern most closely matching that of the cyclohexyl acetate (CA) standard (Fig. 1B and Additional file 2: Fig. S2B). CA is a VSP candidate sharing a similar retention time (Additional file 2: Fig. S2A) and mass spectral features (Fig. 1B and Additional file 2: Fig. S2B) with the natural VSP peak. To improve separation of the isomeric candidates, we used a shallow temperature ramp of 1 °C per min. In Additional file 2: Fig. S2, we tested the top two candidates suggested by mass spectral library matching. The retention time of cis-3-hexenyl acetate differed substantially from the VSP peak, whereas CA exhibited a closely matching retention time (Additional file 2: Fig. S2A). Also, cyclohexyl acetate showed a higher-quality mass spectral match than cis-3-hexenyl acetate in the library comparison. While this does not definitively confirm the identity or structural similarity of the compound, CA received the highest match score among all tested chemical standards in the NIST library.

### Cyclohexyl acetate elicits male-specific attraction

We used the chemoattraction assays adopted from Wan et al. [18, 27] with minor modifications. Chemoattraction assays using 1000-fold diluted CA demonstrated significant attraction in both sexes of *C. elegans* and *C. remanei*, with a robust positive chemotaxis index (Fig. 1C). However, the known VSP chemoreceptor *srd-1* mutant males did not show a significant reduction in chemotaxis toward cyclohexyl acetate, suggesting that detection of this compound does not depend on the SRD-1 chemoreceptor. Sex pheromone blends within a species often consist of mixtures of structurally related compounds [5, 28–32]. For example, the cabbage looper produces a blend in which the major component is (Z)-7-dodecenyl acetate, accompanied by several minor acetate esters [28]. This phenomenon prompted us to screen chemicals that are structural variants of cyclohexyl acetate.

**Fig. 2:**
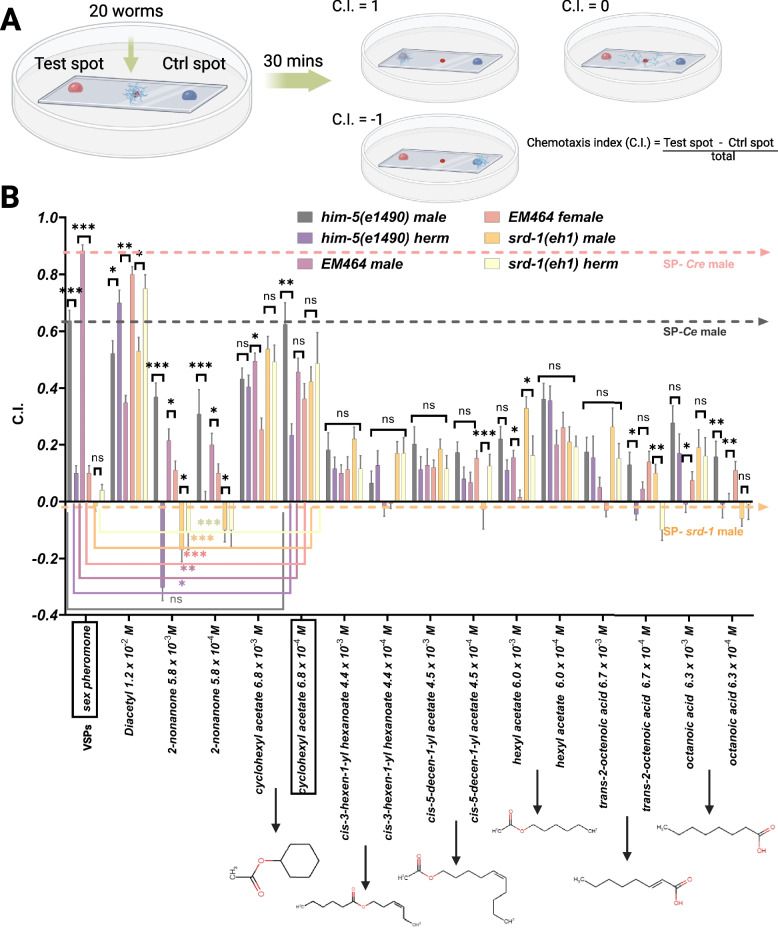
Chemotaxis index of wild-type *C. elegans* and *C. remanei* to VPS and volatile organic compounds at 1:1000 and 1:10,000 dilutions. Chemotaxis indices (C.I.) of *him-5(e1490) C. elegans*, EM464 *C. remanei*, and *srd-1* mutant strain of both sexes in response to different volatile compounds. The graph shows the chemotaxis indices of the test strains in response to VPS and various chemical compounds at different concentrations (1:1000 and 1:10,000), including cyclohexyl acetate, diacetyl acetate, cis-3-hexen-1-yl hexanoate, cis-5-decen-1-yl acetate, hexyl acetate, 2-octenoic acid, and octanoic acid. Positive indices indicate attraction, whereas negative indices indicate repulsion. Diacetyl and 2-nonanone served as the attractant and repellent positive controls, respectively. Sex pheromone (VSPs) served as the positive control. Chemotaxis index (C.I.) = (number of nematodes in the test spot) − (number of nematodes in the control spot) /total number of worms. The error bars represent the standard error. *, **, *** indicate a significant difference (*P* < 0.05, 0.005, and 0.0001, respectively)

Then we evaluated a series of chemoattraction assays using a set of six compounds structurally related to cyclohexyl acetate but varying in hydrocarbon chain length, unsaturation, and the presence or absence of the acetate function group. Both species exhibit similar responses to most odors tested, with the exception of the repellent control 2-nonanone, which elicited a repellent response only in *C. elegans* males. Additionally, cis-3-hexen-1-yl hexanoate, hexyl acetate, trans-2-octenoic acid, and octanoic acid at specific concentrations induced slight attraction in *C. elegans* but not in *C. remanei*. Among those chemicals, CA functions as a strong concentration-dependent male-specific attractant (Fig. 2). EM464 *C. remanei* and CB4088: *him-5(e1490) C. elegans* were used as a standard male-rich reference strain. For *him-5(e1490)* males, CA at a concentration of 6.8 × 10^−3^ M resulted in a chemotaxis index (C.I.) of 0.433 ± 0.038. The response increased at a lower concentration of 6.8 × 10^–4^ M, with a C.I. of 0.625 ± 0.075. This response was comparable to that of the positive control (VSPs, C.I. = 0.637 ± 0.036). In contrast, *him-5(e1490)* hermaphrodites also showed attraction to CA at higher concentrations, with a C.I. of 0.405 ± 0.041 at a concentration of 6.8 × 10^−3^ M, and a decreased response, with a C.I. of 0.233 ± 0.039 at 6.8 × 10^−4^ M. These results confirm that CA is a potent male-specific attractant at certain concentrations. We evaluated the role of SRD-1, a key receptor for VSP [18], via *srd-1(eh1)* mutants (Figs. 1 and 2).

However, the robust response observed in *srd-1* mutants of both sexes, which is equivalent to *him-5* response, suggested that CA detection involves *srd-1*-independent pathways integrated with other neural mechanisms. These data suggest that the unknown compound may share certain fragmentation features with CA, and we therefore interpret CA as a putative structural analog or mimic of a naturally occurring volatile component. Additionally, the observation that CA elicits robust male attraction in VSPs’ major chemoreceptor *srd-1* null mutants suggests that CA may not be a VSP major component; it acts as a structural mimic of natural VSP components and functions via an alternative pathway.

### Cyclohexyl acetate is a concentration-dependent male attractant

**Fig. 3:**
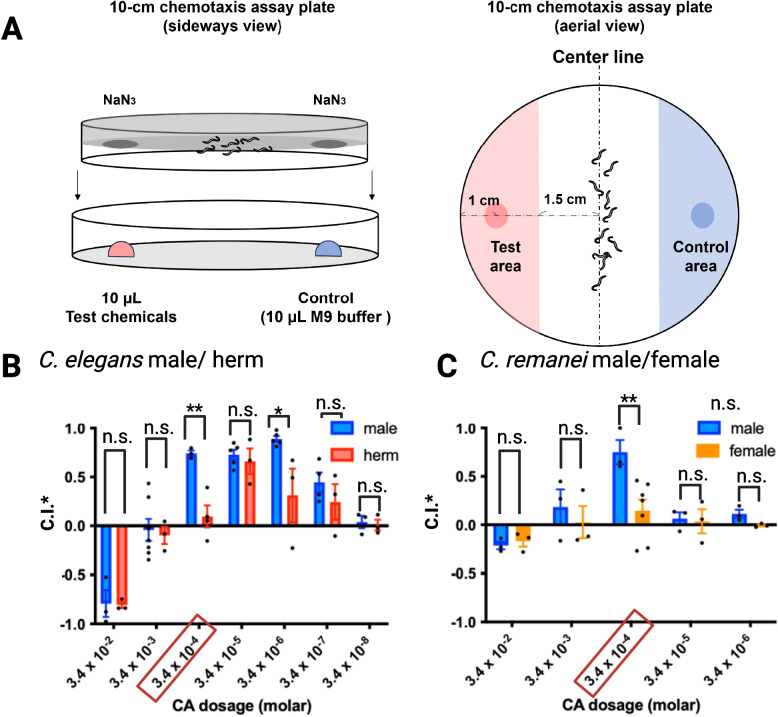
Sex-specific and concentration-dependent responses to cyclohexyl acetate in *C. elegans* and *C. remanei*.** A** Schematic diagram of the 10-cm plate chemotaxis assay. **B** Chemotaxis responses of *C. elegans* males and hermaphrodites across a dilution series of CA from 3.4 × 10^−2^ M to 3.4 × 10^−8^ M. **C** Chemotaxis responses of *C. remanei* males and females across 3.4 × 10^−2^ M to 3.4 × 10.^−6^ M range. Chemotaxis index* (C.I.*) = (number of nematodes in the CA zone) − (number of nematodes in the control zone)/{(number of nematodes in the CA zone) + (number of nematodes in the control zone)}. The error bars indicate the standard error. * and ** indicate a significant difference in the *t*-test (*P* < 0.05 and 0.005, respectively)

To investigate the functional relevance of cyclohexyl acetate (CA) as a candidate mimic of female VSPs, we performed quantitative chemotaxis assays across a broad concentration range, using a protocol adapted from Leighton et al. [12] with minor modifications (see the “Methods” section and Fig. 3A). CA was tested at concentrations spanning seven orders of magnitude (3.4 × 10^−2^ M to 3.4 × 10^−8^ M) to characterize dose-dependent responses in adult *C. elegans* and *C. remanei.*

In *C. elegans*, both males and hermaphrodites displayed concentration-dependent responses to CA, but with distinct behavioral profiles. At high concentrations (3.4 × 10^−2^ M), both sexes showed aversion, suggesting that CA is aversive above a threshold. At intermediate concentrations (3.4 × 10^−4^ M to 3.4 × 10^−7^ M), males exhibited robust attraction, with a chemotaxis index* (C.I.*) peaking near 0.6 (Fig. 3B). Hermaphrodites showed a significantly weaker response at 3.4 × 10^−4^ M and 3.4 × 10^−6^ M. *C. remanei* males also showed significant attraction to CA, with peak responses at 3.4 × 10^−4^ M, whereas *C. remanei* females were not attracted by CA at any concentration tested (Fig. 3C). Together, these findings demonstrate that CA elicits a sex-specific, species-specific, concentration-dependent response, with attraction occurring within an intermediate range and aversion emerging at higher doses.

Although the mechanism underlying aversion to higher concentrations of CA remains unclear, similar concentration-dependent aversions have been well documented for chemicals such as diacetyl, benzaldehyde, and isoamyl alcohol, which are also detected by AWA and AWC neurons [33–35]. At lower concentrations, AWA and AWC neurons trigger attraction, but at higher concentrations, ASH neurons mediate avoidance [33–35].

### Cyclohexyl acetate is perceived by *C. elegans* males via AWC^on^ neurons

Having established that CA is attractive to *C. elegans* and *C. remanei* males, we carried out chemotaxis assays with specific *C. elegans* mutants to identify the neurons responsible for CA perception. Both male-specific neurons (CEMs) and amphid sensory neurons (AWAs) have been implicated in VSP perception in *C. elegans* [15, 17–19]. Accordingly, we assayed males with mutations that affect the function of these neurons as well as that of an additional amphid neuron (AWC) responsible for volatile attractants [5, 17, 36]. Males of the *ceh-36(ks86)* and *njls79 [ceh-36p::cz::casp3, ceh-36p::casp3::nz, ges-1p::GFP](X)* strains, which lack functional AWC neurons [37, 38], were almost completely defective in their response to CA at 3.4 × 10^−6^ M (*p* < 0.0001, Dunnett’s multiple comparison test), whereas males of genotypes *odr-7(ky4)* and *lin-11(n389)*, which lack functional AWA neurons [39, 40], were attracted to the CA at the same level as wild-type (WT) males (Fig. 3A). Males of *ceh-30(n4289)*, which lack functional CEM neurons [41], were slightly less attracted (*p* < 0.05, Dunnett’s multiple comparison test), suggesting that AWC neurons are primarily responsible for CA perception, with a possible contribution from CEM neurons. The two AWC neurons of *C. elegans*, designated AWC^on^ and AWC^off^, display molecular and functional asymmetry, as they sense partly different odors [42]. To determine whether both or only one of the two types of AWC neurons is required for CA perception, we performed chemotaxis assays using mutant lines. Mutant *nsy-4(ky616)* males, which exhibit a two-AWC^off^ phenotype [43], exhibited significantly less attraction (*p* < 0.0001, Dunnett’s multiple comparison test), whereas *nsy-1(ky397)* and *nsy-1(ok593)* mutants, which have two AWC^on^ neurons [44, 45], were attracted to the CA at the same level as WT males having both AWC^on^ and AWC^off^ neurons (Fig. 3B). Therefore, AWC^on^ neurons are primarily responsible for CA perception.

To visualize the response of *C. elegans* AWC^on^ neurons to CA, we used the calcium indicator GCaMP6s for real-time visualization of sensory neuron excitation by monitoring the influx of calcium ions upon exposure to the chemical signal. We generated a transgenic worm strain carrying AWC^on^-specific regulatory sequences (P*str-2* promoter) that directly express the GCaMP6s reporter gene in AWC^on^. Previous studies have demonstrated that the calcium level in AWC^on^ neurons decreases upon odor addition [46–49], and as shown in Fig. 3C and Additional file 4: Fig. S4, the fluorescence intensity in male AWC^on^ neurons largely decreases upon CA stimulation. Specifically, the GCaMP6s signal intensity decreased in the AWC^on^-neuron cell body, dendrites, and axons. The male AWC^on^-neuron GFP intensity decreased immediately after CA stimulation and rapidly decreased within the observation window of 2.5 min. CA is thus sensed by male AWC^on^ neurons. In contrast, hermaphrodites exposed to CA showed a weaker change in AWC^on^ neuron fluorescence intensity; the amplitude was one-fourth that of the male response (Fig. 3D). The signal change was not significant and was more variable than that in the male AWC^on^ neuron response (Fig. 3F). As a control, we tested another AWC-specific odorant, isoamyl alcohol (IAA), and found that the hermaphrodites of the imaging strain can respond to AWC-specific odorants (Fig. 3E). The sexual dimorphism noted in AWC^on^ neuron activity aligns with our previous observations on AWA neurons’ responses to VSP containing natural pheromones [18].

### Cyclohexyl acetate functions as a female/hermaphrodite volatile sex pheromone mimic in *C. elegans*

To support our hypothesis that CA mimics a functional component of the VSPs produced by *C. elegans* hermaphrodites, we conducted adaptation assays with *C. elegans* males to the VSP extract from *fog-2* females, which contains the native VSPs [12], and to CA specifically (Fig. 5). If prior exposure to VSPs does not reduce the male response to CA, it would suggest that CA is not mimicking VSPs. Conversely, if VSP exposure diminishes the response to CA, it would support the idea that CA functions as a mimic of VSPs. The observed reduction in chemotactic response to CA following VSP exposure serves to evaluate whether CA functions as a structural and functional mimic of the natural VSPs.

Initial exposure to both the natural VSP extract and CA strongly attracted male subjects, thus verifying their attractiveness to males in this behavioral assay context (Fig. 5). Pre-exposure to both samples for 10 min significantly reduced male responsiveness, indicating olfactory adaptation to both the pheromone and the same concentration of CA, as evidenced in Figs. 5B, C, and G. These behavioral experiments revealed that variations in odor preferences arise from pheromone-specific adaptation, suggesting that pre-exposure results in the rewiring of pheromone perception neural circuits. Not all odorants induce adaptation; for example, males pre-exposed to diacetyl (2,3-butanedione, DA) remain attracted to the same concentration upon re-exposure (Additional file 5: Fig. S5C).

To further explore a possible functional role of CA or a CA analog as a VSP mimic, we conducted an experiment where males were first exposed to the natural VSPs for 10 min and then assessed for their chemotactic behavior toward CA. The underlying hypothesis is that if a chemical induces adaptation following pre-exposure and if this chemical is a constituent of a mixture, then prior exposure to this mixture should result in a reduced chemotactic response to this chemical, signaling olfactory adaptation, a behavior that has been demonstrated in *C. elegans* [49]. The results supported this hypothesis: pre-exposure to either the natural VSPs or CA led to a suppression of the chemotactic response to CA (Fig. 5C, E, and G). We observed that males typically need approximately 16–20 min to respond robustly to CA, in contrast to the peak responses to other tested stimuli, which occur within 6–10 min. Therefore, the duration of the initial exposure was increased by an extra 10 min (Fig. 5C). Males pre-exposed to CA retained attraction to the full VSP blend (Fig. S5A). This indicates that CA may specifically mimic only one component of the VSP mixture. Sensory adaptation to CA selectively affects responses to some structural analogs, but not the entire VSP mixture.

As a structurally analogous control to CA, 2-cyclohexylethanol (2CH) attracts males via AWC neurons [48] but is absent from natural VSP extracts. 2CH shows completely distinct mass spectral signature fragments to VSPs. The fragmentation profile of the 2CH standard is missing the diagnostic m/z 43 fragment (characteristic of CA and natural VSP components), and it also has additional fragments at m/z 41, 55, 68, 69, 81, and 110. These spectral signatures were never detected in VSP GC peak, confirming 2CH’s absence from the natural pheromone blend. We also performed a serial dilution assay and found that 2CH was more attractive to males at 1.4 × 10^−3^ M than at 3.4 × 10^−4^ M (Additional file 3: Fig. S3A). Therefore, we selected 1.4 × 10^−3^ M for the adaptation assay, as it elicited the most robust attraction response in males (Additional file 3: Fig. S3A). We also tested hermaphrodite responses to different concentrations of 2CH. Hermaphrodites were more attracted to higher concentrations but exhibited a repulsive response at the lowest concentration tested (3.4 × 10^−6^ M). Upon two exposures, 2CH did not produce a typical adaptation pattern. Instead, males are highly attracted by 2CH and remain predominantly at the site of initial exposure, resulting in limited migration to the alternate region and a minimal increase in the “other” zone. These dynamics indicate that 2CH induces a strong site-retention effect (Fig. 5D). Pre-exposure to VSP partially reduced the chemotactic response to 2CH (Fig. 5F), though this attenuation was much less pronounced than the near-complete suppression observed for CA.

Functionally, CA by itself fails to reproduce the full VSP-evoked attraction, while SRD-1–dependent responses remain strong for VSPs. Thus, an alternative to a CA-like analog/mimic (Fig. 1B) is that the physiological responses require CA plus additional VSP components sensed via SRD-1, with CA constituting one part of the mixture.

## Discussion

### Cyclohexyl acetate mimics a nematode sex pheromone to drive odor-specific olfactory adaptation via AWC neurons

This study identifies CA as a potent mimic of the natural *C. elegans* VSP and demonstrates that pre-exposure to CA or the natural pheromone mixture induces odor-specific adaptation in males. This study also demonstrated that AWC neurons mediate odor-selective adaptation rather than generalized sensory desensitization. These results show the specificity of chemosensory adaptation, allowing males to dynamically adjust their responses based on recent odor exposure. For example, pre-exposure to certain chemicals, such as 2CH, causes males to remain at the original exposure site. In contrast, pre-exposure to VSPs or CA induces adaptation, leading to a diminished response to subsequent exposures at the same concentration. Other chemicals, such as diacetyl, do not produce either retention or adaptation effects (Figs. 5 and S5).

Notably, the CA mimics only a subset of the effects of natural pheromones. Unlike the full VSP, the CA does not rely on AWA neurons or the SRD-1 receptor for detection [18], suggesting that it replicates a single component of the multi-odorant pheromone system but is itself not a functional component of the VSP [18]. This highlights the intricacy of the *C. elegans* chemical communication system, where multiple pheromone components likely act in synergy to elicit the full behavioral response [2, 32, 50]. The identification of CA raises questions about its biological origin: while its role as a pheromone mimic is clear, it remains unresolved whether CA is an endogenous product of *C. elegans*, a microbial metabolite, or an environmental compound. This ambiguity highlights the need to investigate the natural occurrence and ecological relevance of CA, particularly given its potential to act as a decoy signal in habitats where microbes or other nematodes might produce structurally similar volatiles.

CA triggered variable GCaMP6s signal excitation in some hermaphrodite AWA neurons, whereas, in males, it elicited a steadier and stronger GCaMP6s signal response. These results are similar to those we previously reported for VSPs [19]: VSPs elicited an unstable, receptor-dependent excitation of the GCaMP6s signal in hermaphrodite AWA neurons, whereas, in male AWA neurons, the VSP evoked a more stable receptor-dependent GCaMP6s signal with a larger amplitude.

### Potential implications for cross-species interactions

While our study demonstrates that CA elicits male-specific attraction in *C. elegans* and *C. remanei*, its broader ecological significance remains to be explored. The observed difference in behavioral responses between *C. elegans* hermaphrodites and *C. remanei* females may reflect underlying differences in their reproductive strategies. *C. remanei,* a dioecious species, is subject to stronger sexual selection pressures that likely favor the evolution of strict sex-specific signaling mechanisms. In contrast, *C. elegans* is androdioecious, and hermaphrodites rarely outcross, reducing the selective pressure for female-specific pheromone responses.

The structural resemblance of CA to known nematode signaling molecules raises questions about potential cross-species interactions, as observed in other systems. For instance, nematophagous fungi exploit nematode-derived ascarosides to trigger trap formation, and plant-parasitic nematodes use pheromonal cues to facilitate beetle-mediated dispersal [25]. By analogy to methyl 3-methylbutanoate (MMB), which is both a component of nematode male pheromone and an attractive mimic produced by the nematophagous fungus *Arthrobotrys oligospora* to lure its prey [49, 51], our identification of cyclohexyl acetate (CA) as a potent, female-specific attractant suggests that CA could likewise function as an ecologically volatile cue that may be co-opted by other species. If CA or structurally similar compounds are present in natural environments, they could be co-opted by predators as decoy signals or by symbiotic microbes to modulate host behavior. However, these scenarios remain speculative and require validation in ecologically relevant contexts. Notably, the endogenous source of CA has yet to be identified. To address these gaps, future studies should investigate whether CA is produced by soil-dwelling microbes or nematodes, assess its effects on non-*Caenorhabditis* species, and characterize the chemical composition of natural nematode habitats. Such efforts will be essential to clarify the ecological roles and evolutionary implications of CA in nematode communication.

### Role of *srd-1* in mediating male attraction to volatile cues

The *srd-1(eh1)* males presented a slightly decreased attraction response to CA (*him-5(e1490)* males, C.I.* = 0.628 ± 0.075; *srd-1(eh1)* males, 0.423 ± 0.052; at 6.8 × 10^−4^ M). A similar level of reduction was observed in response to other compounds, such as octanoic acid (*him-5(e1490)* males, C.I. = 0.278 ± 0.060*; srd-1(eh1)* males, C.I.* = 0.190 ± 0.063; at 6.8 × 10^−3^ M); cis-5-decen-1-yl-acetate (*him-5(e1490)* males, C.I. = 0.173 ± 0.037*; srd-1(eh1)* males, C.I.* =  − 0.028 ± 0.069; at 6.8 × 10^−4^ M dilution); and cis-3-hexenyl hexanoate (*him-5(e1490)* males, C.I. = 0.182 ± 0.060*; srd-1(eh1)* males, C.I.* = 0.022 ± 0.042; at 6.8 × 10^−3^ M), among others. The consistent slight reduction in the chemotaxis index observed in *srd-1(eh1)* mutants across multiple volatile candidate derivatives suggests that *srd-1* specifically affects the detection of VSP structurally similar components. This pattern aligns with previous studies showing that *srd-1* mutants retain normal chemotaxis responses to pheromone-unrelated AWA-sensed volatile attractants [18], indicating that *srd-1* mutants do not have general defects in chemotaxis or mobility. This finding suggests a specific role for *srd-1* in recognizing particular chemical structures.

Overall, CA provides a fascinating lens for studying the complexity of sex pheromone communication in nematodes. While CA mimics a component of the *C. elegans* female VSP, its possible biogenic sources and potential for cross-species exploitation suggest critical questions for future research. These findings lay the groundwork for unraveling the ecological and evolutionary mechanisms driving volatile signaling in nematodes and their interactions with other organisms in their environment.

## Conclusion

CA elicits robust, concentration-dependent attraction in *Caenorhabditis* males and recapitulates key behavioral features of the natural VSP mixture, including odor-specific adaptation following pre-exposure. Genetic and neural analyses indicate that CA perception is mediated primarily by AWC_on_ (*str-2*-expressing) neurons and proceeds largely through an *srd-1*-independent pathway, distinguishing CA sensing from the SRD-1/AWA-dependent processing of natural VSPs. Together, these results support the view that CA is unlikely to be a major endogenous VSP constituent; rather, it functions as a structural analog/mimic that engages a parallel chemosensory circuit capable of driving male-specific attraction and adaptation. An important next step is to identify the endogenous source of CA-like activity and determine whether CA is produced by nematodes, associated microbes, or environmental substrates, and whether natural VSP responses require CA in combination with additional SRD-1-dependent components.

## Methods

### Volatile compound collection for GC–MS

The mated *C. remanei* (wild-type EM464) female loses the ability to produce VSPs [12]. Males and females can be reliably distinguished based on morphology beginning in the L4 larval stage. To obtain virgin females for experiments, L4-stage animals were isolated from mixed populations. The operational window for virgin female collection spans approximately 15–16 h. To extend this period and obtain sufficient numbers, L1-arrested worms were released at three time points spaced 2 h apart, expanding the collection window to 20 h. The following day, virgin females were confirmed by the absence of eggs on the plate.

A total of 5000 1-day-old *C. remanei* were collected for each sample and washed three times with M9 buffer. Volatile components were isolated using a purge-and-trap system with a Tenax TA adsorbent liner (poly(2,6-diphenyl-p-phenylene oxide), 60–80 mesh) [52]. Tenax TA selectively traps volatile and semi-volatile analytes within a molecular range of n–C₇ to n–C₂₆. As illustrated in Additional file 3: Fig. S3, samples (2 mL) were loaded into a glass vial sealed with a rubber stopper and two gas-flow needles. The vial was placed in a heated water bath (50 °C), and volatile compounds were purged under a nitrogen stream (99.999% purity) at 30 mL/min for 10 min. The volatiles were carried by the gas flow and trapped onto the Tenax TA adsorbent. To minimize thermal degradation and potential analyte loss, the entire purge-and-trap assembly was maintained at 4 °C, ensuring rapid cooling of outflowing gases from the glass vial. Post-collection, the Tenax TA liner was directly transferred to a gas chromatography-mass spectrometry (GC–MS) system (Agilent) for thermal desorption and analysis (see Additional file 3: Fig. S3B).

### GC–MS analysis

Data present in Fig. 1, volatile compounds were analyzed using GC–MS equipped with a DB-17 ms capillary column (30 m × 0.32 mm). The inlet temperature was set to 280 °C. The GC temperature program consisted of an initial hold at 40 °C for 2 min, followed by a temperature ramp from 40 °C to 130 °C at 5 °C/min (Segment 1), then from 130 °C to 250 °C at 20 °C/min (Segment 2), with a final hold at 250 °C for 3 min (Segment 3). Helium was used as the carrier gas at a constant column flow rate of 1 mL/min. MS acquisition was performed using the standard spectra tuning mode. Mass spectral data were analyzed using the National Institute of Standards and Technology (NIST) library.

Data present in Additional file 1 and 2: Fig. S1 and S2, volatiles from female and hermaphrodite cultures were collected by headspace solid-phase microextraction (SPME). A DVB/CAR/PDMS fiber (50/30 µm; Supelco, Bellefonte, PA) was preconditioned in the GC injector at 265 °C for 10 min before each use. For sampling, the fiber was exposed to the headspace of sealed culture vials at 60 °C for 10 min under continuous agitation (250 rpm; 5 s on/20 s off) using a CTC Analytics autosampler. Following extraction, the fiber was immediately desorbed in the GC injector for 6 min. Analyses were carried out on an Agilent 6890N gas chromatograph coupled to a Waters GCT Premier orthogonal-acceleration TOF mass spectrometer. Analytes were separated on a DB-624 capillary column (30 m × 250 µm × 1.40 µm; Agilent) with helium carrier gas at a constant 0.80 mL min^−1^. The oven was held at 25 °C (0 min), ramped at 5 °C min^−1^ to 85 °C (0 min), then at 1 °C min^−1^ to 150 °C (0 min), and finally at 30 °C min^−1^ to 250 °C (1.5 min hold). Spectra were acquired under electron ionization (70 eV) in positive-ion mode across m/z 35–500. Blank runs (SPME fiber exposed to Milli-Q H₂O) were interleaved with samples to monitor carryover. Instrument performance was verified daily using alkane standards (C7–C30).

### Sample preparation for chemotaxis assays

Worm-conditioned media, the native VSPs from *C. elegans* hermaphrodites and *C. remanei* females in M9 buffer were prepared and used for the chemotaxis assay according to the methods of Leighton et al. [12] and Wan et al. [27]. For the initial chemical screens (Figs. 1 and 2), test compounds were prepared at 1000-fold and 10,000-fold dilutions. Both the 1000-fold and 10,000-fold dilutions were prepared to ensure that the final ethanol concentration remained constant at 0.01%. Control solutions were prepared using the same ethanol and M9 buffer ratios, but without the test compound.

**Fig. 4:**
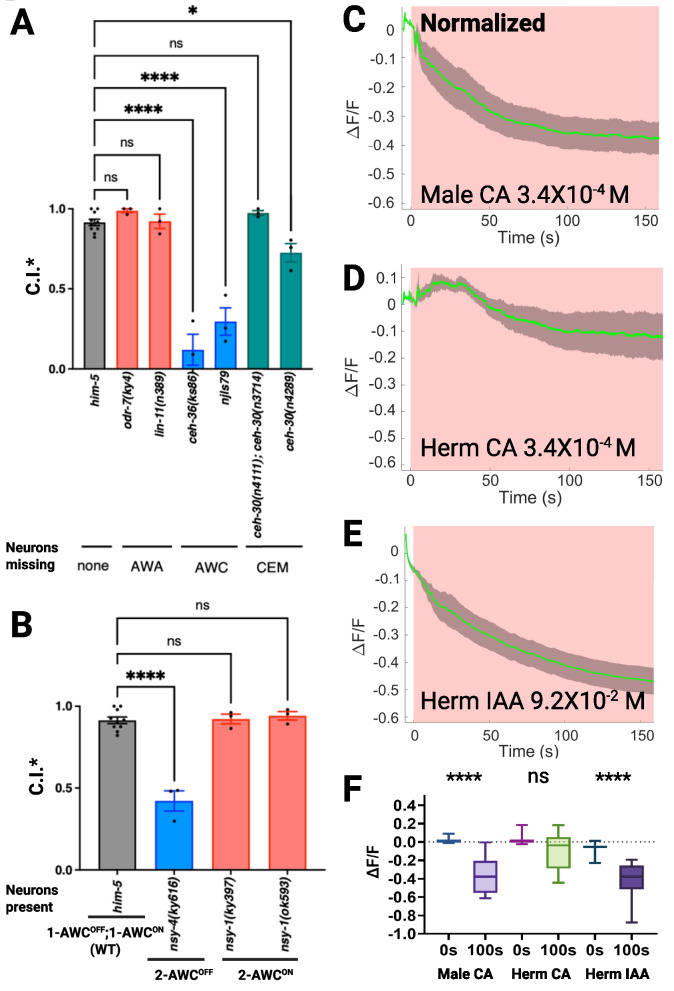
Chemotaxis deficits in *C. elegans* pheromone-detecting neuron mutants and sex-dimorphic AWC_on_ neuronal activity in response to cyclohexyl acetate. **A** Male attraction of *C. elegans* AWA, AWC, and CEM neuron-defective mutants to CA at 3.4 × 10^−6^ M. **B** Male attraction of *C. elegans* 2-AWC^on^ and 2-AWC^OFF^ mutants to CA at 3.4 × 10^−6^ M. Error bars indicate standard error. *, **** indicate a significant difference (*P* < 0.05 and 0.0001, respectively) in C.I. between the mutants and *him-5* control based on Dunnett’s test. **C** to **E** Activity of AWC^on^ neurons in *C. elegans* adult males or hermaphrodites (herm) in response to CA and IAA. GCaMP6s signals were normalized to coelomocyte GFP to correct for eliminated photobleaching effect, which was measured via the same setup. Green line: ∆F/F average. Gray shading: s.e.m. envelope. The red bar indicates the stimulation time. *n* = 14 and 9 for the CA test in males and hermaphrodites, respectively, and *n* = 15 for the IAA-positive control. **(F)** GCaMP6s signal intensity of *C. elegans* males and hermaphrodites subjected to CA stimulation and hermaphrodites subjected to IAA stimulation at 0 s and 100 s after stimulus delivery

**Fig. 5:**
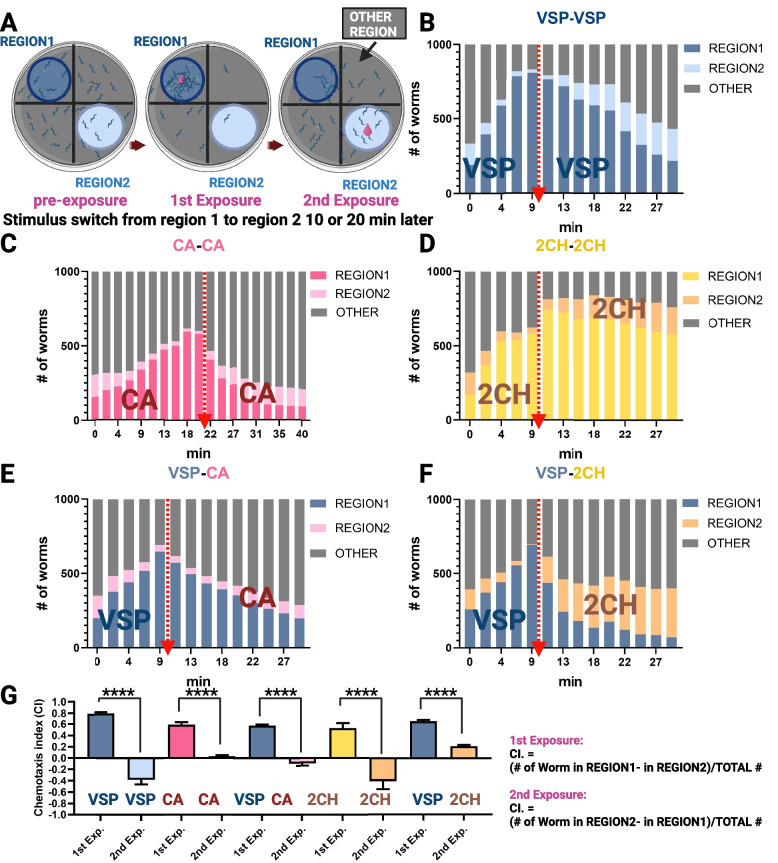
Adaptation assay schematic and stimulus-specific attenuation of male chemotaxis in *C. elegans* to VSP media and volatile compounds with cross-adaptation effects. **A** A schematic overview of the adaptation assay procedure. One-day-old adult *him-5* males were transferred to assay plates and were allowed to distribute for 10 min. Sequential stimuli (VSP, CA, or 2CH) were applied to designated regions. First, apply the stimulus to Region 1. Then, remove the stimulus from Region 1 and apply the second stimulus to Region 2. WormLab software recorded and quantified worm positions at 2.22-min intervals. **B**, **C** Males pre-exposed to worm-conditioned VSP media or CA are no longer attracted by the same concentration of VSP or CA, respectively. **D** Males pre-exposed to 2CH are not attracted by the same concentration of 2CH in Region 2. **E** CA in the second exposure test no longer attracted males that were pre-exposed to VSP. **F** 2CH still attracted males that were pre-exposed to SP. **G** Chemoattraction index was measured after the first and second exposures. Significance was determined by a paired *t*-test: ****P < 0.0001. The error bars indicate the SDs. The CA concentration is 3.4 × 10^−4^ M, and the 2CH concentration is 1.4 × 10^−3^ M. The dashed line with an arrowhead indicates the chemical switch

For the concentration–response, mutant assays, and adaptation assay (Figs. 3, 4, 5, and S3), a serial dilution series of cyclohexyl acetate (CA) and 2-cyclohexen-1-ol (2CH) was prepared, starting from a 50% (v/v) stock solution in ethanol. Serial dilutions were made in M9 buffer to generate seven final concentrations ranging from 3.4 × 10^−2^ M to 3.4 × 10^−8^ M.

### Chemotaxis assays

Chemotaxis assays were performed according to Leighton et al. [53] and Wan et al. [19, 28] with slight modifications. Both the health and age of the worms affect their behavioral response to pheromones. The worms used in the chemoattraction assay were first bleached and synchronized to ensure that they were clean and at the same developmental stage. Worms were separated from individuals of the opposite sex by picking during the L4 stage and were then fed for another day. The worms were then rinsed from the plate with M9 and then washed twice with M9 buffer and then twice with ddH2O. The chemotaxis assay was carried out at room temperature in a 6- or 10-cm Petri dish prepared with chemotaxis media (2.0% agar, 1 mM MgSO4, 1 mM CaCl2, and 25 mM KH2PO4 (pH 6.0)).

For the chemical screen (Figs. 1 and 2), we adapted a 6-cm plate assay system. A glass coverslip pre-coated with chemotaxis media agar was placed in a Petri dish. The experimental site added 2 μL of test compound (e.g., VSPs) and 2 μL of 1 M sodium azide on the agar surface. Sodium azide was used to immobilize worms upon arrival. The control site added 2 μL of control buffer and 2 μL of 1 M sodium azide on the plate. For 6-cm Petri dishes, the standard distance between the center and each test site was set to 1.5 cm. To initiate the assay, 20 healthy, actively moving worms were manually picked and released simultaneously at the center point. Immediately afterward, 2 μL of the test compound and control solution were added to the corresponding spots. The plate was gently closed and placed undisturbed in a room temperature-stable environment. After 30 min, the number of worms at each site was counted. Worm picking was completed within 1–2 min, and the entire setup process was finished within 5 min to ensure assay consistency.

For chemical concentration–response and mutant assays (Figs. 3 and 4), a modified lid-based chemotaxis protocol was used. Approximately 100 worms were pipetted directly onto the center of each chemotaxis plate. Two 1-μL drops of 1 M sodium azide were applied to opposite sides of the agar surface. A 100-μL aliquot of cyclohexyl acetate (CA) at concentrations ranging from 3.4 × 10^−2^ M to 3.4 × 10^−8^ M was placed on the inner lid above the sodium azide spot. For VSP assays, 10 μL of VSP extract was applied to the lid above the sodium azide site, with 10 μL of M9 buffer used as a control above the opposite sodium azide site (Additional file 3: Fig. S3). Plates were incubated for 30 min before scoring, and chemotaxis indices were calculated based on worm distribution. The assay plate was then placed in a small box to eliminate the influence of light. The assay was run for at least 1 h. If > 20% of the nematodes were still alive after 1 h, we extended the incubation time until > 80% of the nematodes tested died, as determined by periodic checking. This took between 1 and 4 h. For Fig. 3A and B, the *him-5(e1490)* control strain was tested in 10 independent biological replicates, while all other neuron-defective mutants were each tested in three independent biological replicates. Each replicate represents an independent plate assay with worms cultured separately on different plates.

### Calcium imaging

Intracellular calcium signals were measured by monitoring Ca^2+^ binding to a calcium indicator, GCaMP6s; this binding increases the intensity of green fluorescence in cells. Calcium imaging was performed in a disposable worm-mounting and chemical-loading device, the thin-layer microdiffusion (TLMD) unit [18]. For the neural-excitation assays, we used a Nikon STORM spinning-disc microscopy system, which comprised a Nikon Ti2-E motorized inverted microscope, a Yokogawa CSU-X1 spinning disk with microlenses, and two EMCCD cameras for detection. A Nikon 40X dry objective lens was used. Time-lapse images were acquired at ~ 8 frames/s for 150 s at a 488 Hz laser intensity of 20%. The test samples were added manually at ~ 5 s after filming started. Data analysis and video stacking were performed in ImageJ. Both AWC-neuron cell-body and dendrite regions near the cell-body were selected and analyzed. Coelomocyte GFP serves as an internal control for photobleaching correction. The normalized GCaMP6s signal intensity was calculated by normalizing the recorded intensity at each time point against the average coelomocyte GFP fluorescence intensity photobleach rate measured by the same setup. The fluorescence changes were normalized to the pre-stimulation background (before time 0), and the relative intensity values ∆F/F are presented here. The data is presented as the means ± s.e.m. All the data were analyzed with GraphPad Prism 6 and Microsoft Excel. Figures were plotted via custom MATLAB code [53]. Green line: ∆F/F average. Gray shading: s.e.m. envelope. The red or yellow bar indicates the stimulation time.

### Adaptation assay in *C. elegans*

Before the start of the assay, all of the one-day-old adult worms of CB4088*: him-5(e1490)* (standard male-rich reference strain) to be tested were washed from the OP50 seeded plate with M9 buffer and transferred to a clean plate without food. This was to ensure that all bacteria were removed from the body surface of the worms so that the worms would not be affected by the attraction of food during the assay. Before the test, the worms were examined via a chemoattraction assay with a positive control: 1.2 × 10^−2^ M (1:1000) diacetyl (a well-documented *C. elegans* attractant diluted in 10% ethanol) and VSP extracted from *C. elegans fog-2* virgin adult females. Only the worms that passed this quality test were used for further examination.

A 9-cm Petri dish was prepared with 48 mL of adaptation assay agar (1.5% agar, 25 mM NaCl, 1.5 mM Tris-base, and 3.5 mM Tris–Cl). A circle of radius 1 inch was marked in the center of the Petri dish and divided into four quadrants, with two stimulus regions delineated at the centers of two opposite quadrants (labeled regions 1 and 2). All the markings were marked on the bottom of the Petri dish. A group of 100 synchronized and M9 precleaned worms was placed in the center starting site of the Petri dish and allowed to be distributed on the plate over a 10-min period.

In the first stimulus exposure step, a 2-µl aliquot of VSP from *C. elegans fog-2* females, 3.4 × 10^−4^ M (1:20,000) cyclohexyl acetate (CA) or 1.4 × 10^−3^ M (1:5000) 2-cyclohexylethanol (2CH) solution, was placed on the lid corresponding to the center point of region 1. The lid was closed, and the assay was recorded via WormLab (MBF Bioscience). At the 10- or 20-min mark, the stimuli on the lid of Region 1 were removed, and the second exposure step was initiated by placing the second stimulus sample on the lid of the center point of Region 2. The recording continued for 20 min. WormLab software was used to perform data analysis, with the number of worms located within regions 1 and 2 counted every 1000 frames equating to approximately 2.22 min, where the frame rate was 7.5 fps. We administered the first exposure solution and conducted recordings for either 10 or 20 min, corresponding to either 4500 or 9000 frames. This resulted in 5 and 10 data points (including T0), respectively. Subsequently, we temporarily paused the recording for several seconds to switch the exposure solution. Upon restarting the recording following the introduction of the second exposure solution, we continued the recording for an additional 20 or 30 min, equivalent to 9000 or 13,500 frames, thereby yielding 9 and 14 data points, respectively. In the VSP adaptation assay (Additional file 5: Fig. S5B), the duration of the second exposure was prolonged to 30 min. This additional time was vital for determining the point at which no further significant changes in the chemoattraction index were noted, guiding the timing for subsequent experiments.

The chemotaxis index (C.I.) was calculated as the difference between the number of worms in the experimental region and the control region divided by the total number of tested worms. A C.I. value of 1.0 indicates that all the worms are attracted to the experimental spot, and a C.I. value near zero means that the test material is not attractive to the worms. Assays were performed on three separate days with freshly prepared samples. The sample size was 10 assays (1000 animals) for each test.

## Supplementary Information

Additional file 1: Figure S1. GC–MS extracted ion chromatograms (EICs) at m/z 82.0 comparing pheromone-positive (1-day-old unmated C. remanei EM464 females) and pheromone-negative (1-day-old C. remanei EM464 mixed-population and unmated males) samples.

Additional file 2: Figure S2. GC–MS EICs and representative mass spectra showing the VSP peak and the cyclohexyl acetate and cis-3-hexenyl acetate peaks, with conserved retention times and spectra across samples.

Additional file 3: Figure S3. Chemotaxis assay design and dose-dependent response to 2CH in C. elegans; schematic of the GC–MS sample collection (purge-and-trap) system.

Additional file 4: Figure S4. Cyclohexyl acetate–evoked calcium responses in male AWCon neurons (non-normalized mean ± s.e.m., individual traces, and raw traces).

Additional file 5: Figure S5. Pre-exposure/adaptation assays showing attraction after CA pre-exposure, loss of attraction after VSP pre-exposure, and persistence of attraction after diacetyl pre-exposure; timing of chemical switch indicated.

## Data Availability

No datasets were generated or analysed during the current study.

## Abbreviations

2CH: 2-Cyclohexylethanol
AWA: Amphid wing “A” olfactory neuron
AWC: Amphid wing “C” olfactory neuron
AWC_on_: AWC_on_ (*str-2*-expressing AWC subtype)
AWC_off_: AWC_off_ (no *str-2*-expressing AWC subtype)
CA: Cyclohexyl acetate
*C. elegans*: *Caenorhabditis elegans*
*C. remanei*: *Caenorhabditis remanei*
CEM: Cephalic male neuron(s)
CI: Chemotaxis index
EIC: Extracted ion chromatogram
fps: Frames per second
GC–MS: Gas chromatography-mass spectrometry
GC–TOFMS: Gas chromatography-time-of-flight mass spectrometry
GCaMP6s: Genetically encoded calcium indicator GCaMP6s
GFP: Green fluorescent protein
IAA: Isoamyl alcohol
M9: M9 buffer
MMB: Methyl 3-methylbutanoate
NIST: National Institute of Standards and Technology
SD: Standard deviation
s.e.m.: Standard error of the mean
SPME: Solid-phase microextraction
TLMD: Thin-layer microdiffusion device
VSPs: Volatile sex pheromones
WT: Wild type
