# Supplementary figures and images for: Cyclohexyl acetate functions like a volatile sex pheromone mimic in Caenorhabditis nematodes
Source: BMC Biol. 2026 Jan 15;24:34. doi: 10.1186/s12915-026-02510-0 (PMC12892764; doi:10.1186/s12915-026-02510-0)

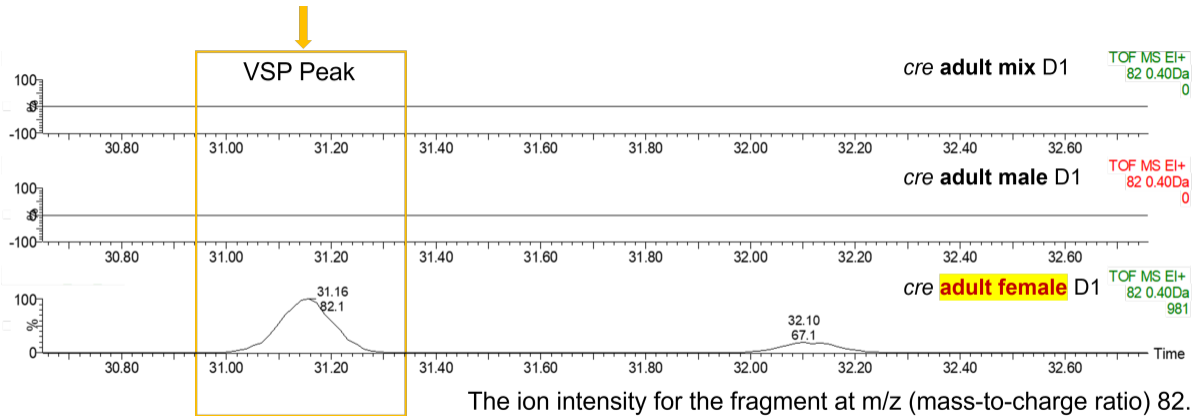

Supplement: Supplementary file 1 — Additional file 1: Figure S1. GC–MS extracted ion chromatograms (EICs) at m/z 82.0 comparing pheromone-positive (1-day-old unmated C. remanei EM464 females) and pheromone-negative (1-day-old C. remanei EM464 mixed-population and unmated males) samples. [file 12915_2026_2510_MOESM1_ESM.pdf]

# A

## cyclohexyl acetate and cis-3 hexenyl acetate 1 $\mu$ M\_GC

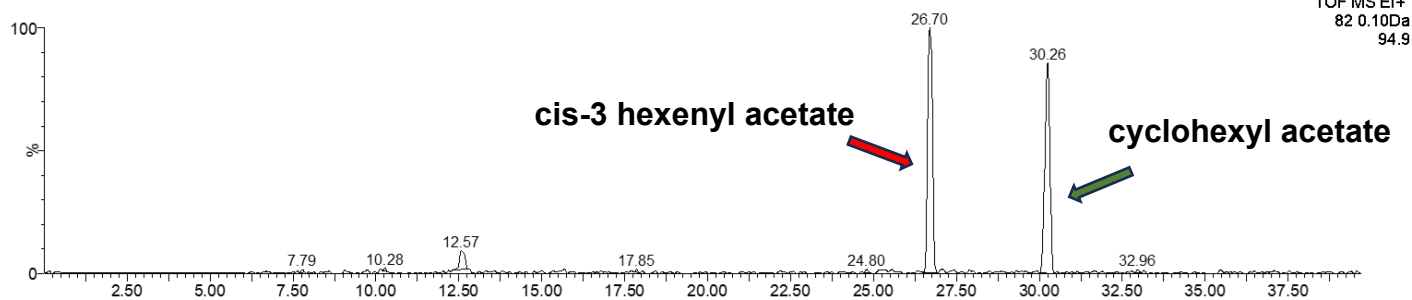

## Cre adult female D1\_GC

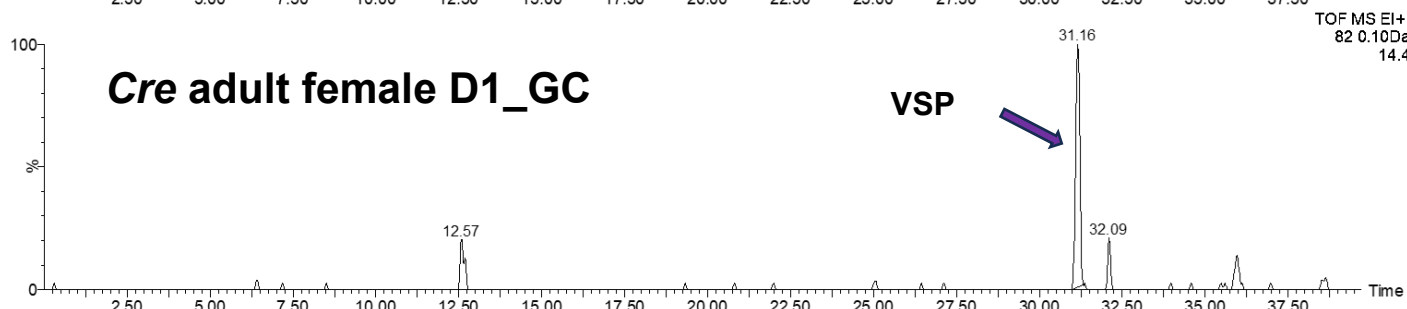

# B

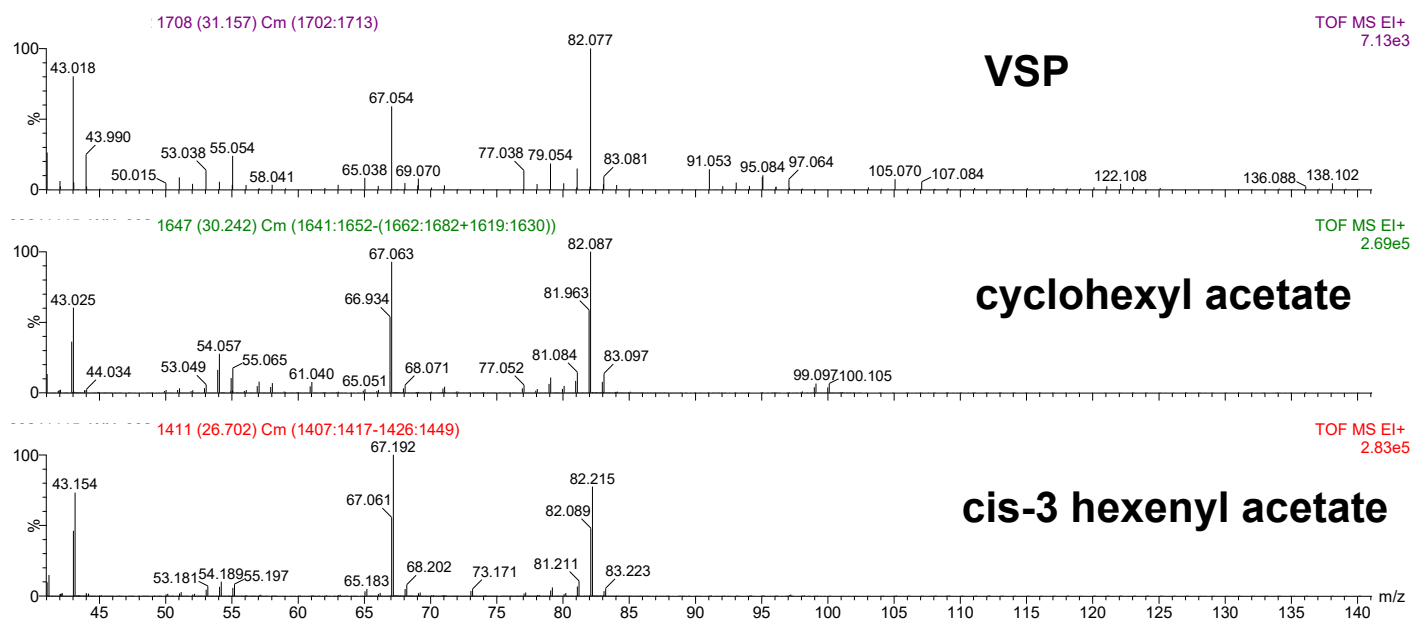

Supplement: Supplementary file 2 — Additional file 2: Figure S2. GC–MS EICs and representative mass spectra showing the VSP peak and the cyclohexyl acetate and cis-3-hexenyl acetate peaks, with conserved retention times and spectra across samples. [file 12915_2026_2510_MOESM2_ESM.pdf]

**A**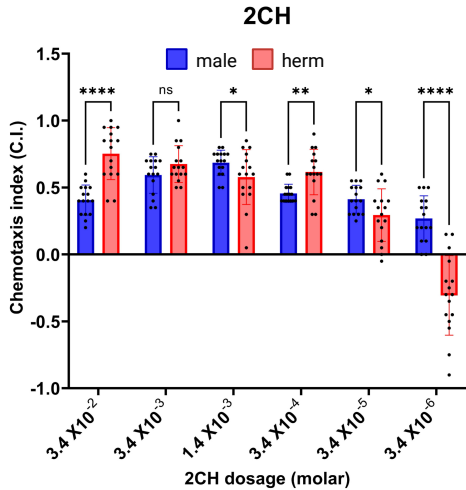**B**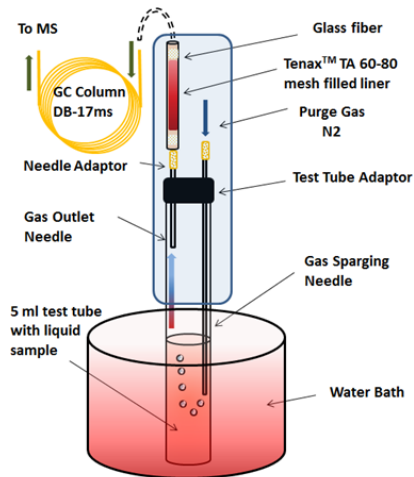

Supplement: Supplementary file 3 — Additional file 3: Figure S3. Chemotaxis assay design and dose-dependent response to 2CH in C. elegans; schematic of the GC–MS sample collection (purge-and-trap) system. [file 12915_2026_2510_MOESM3_ESM.pdf]

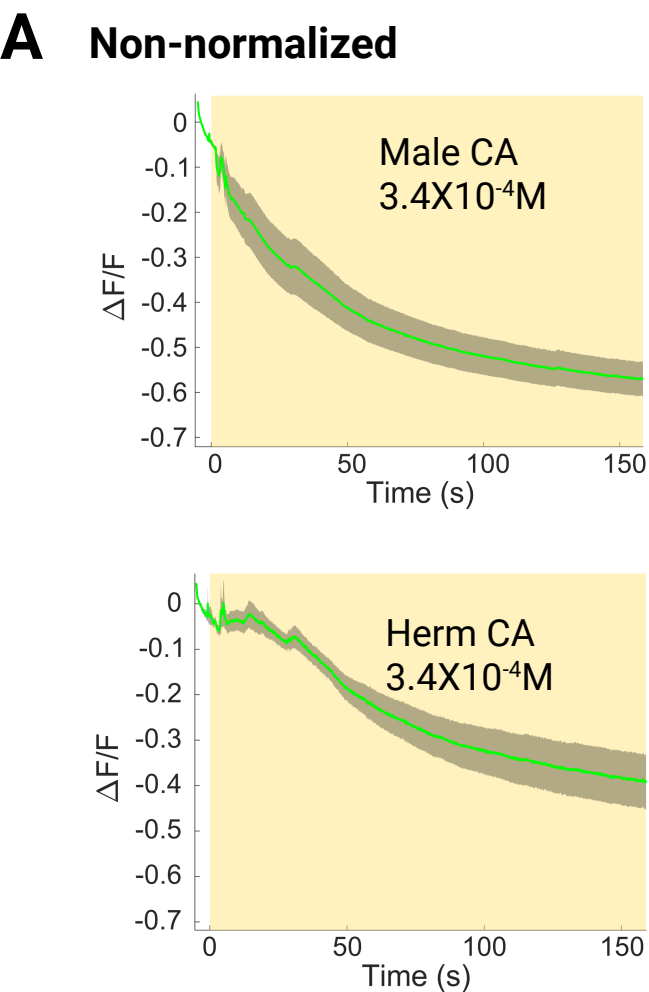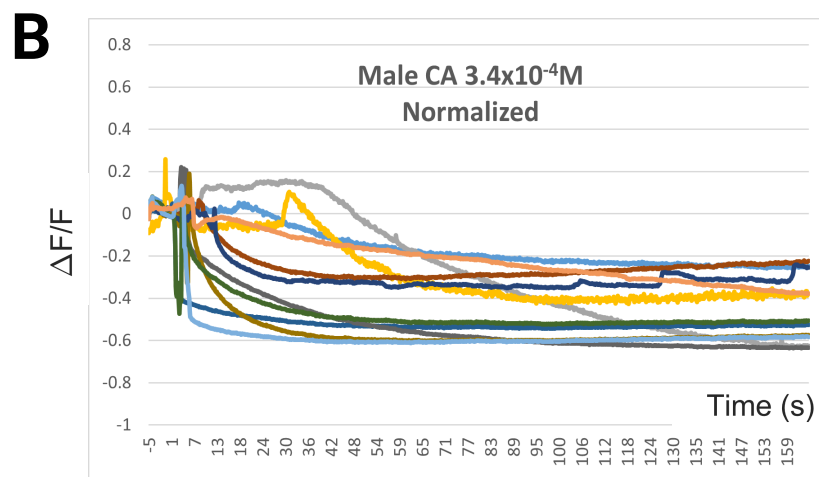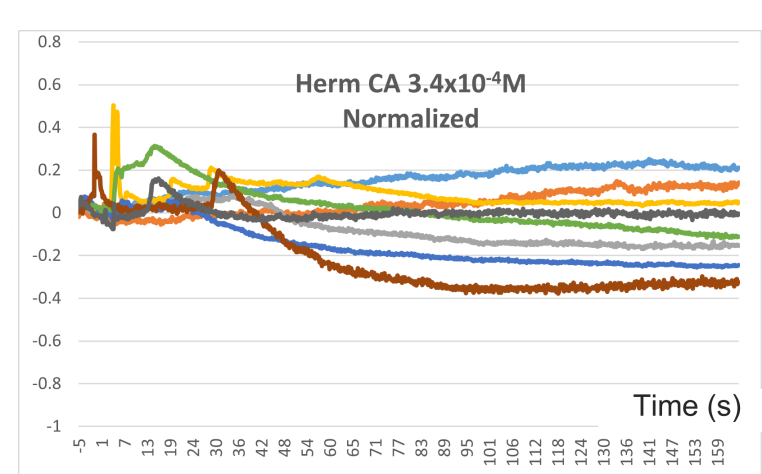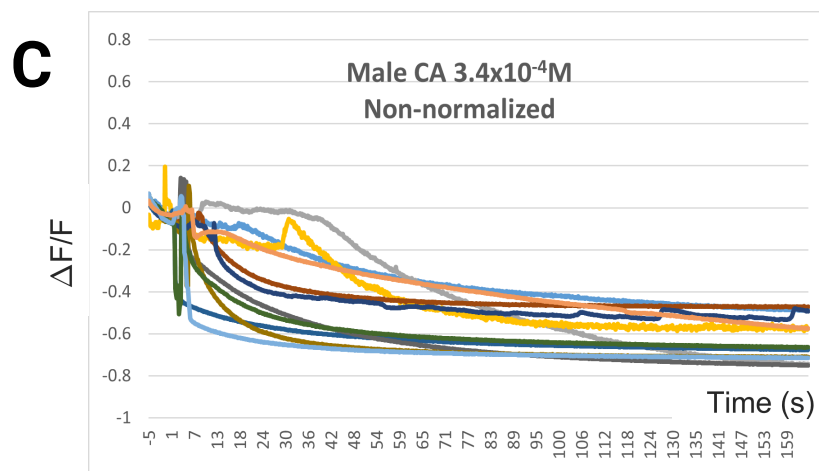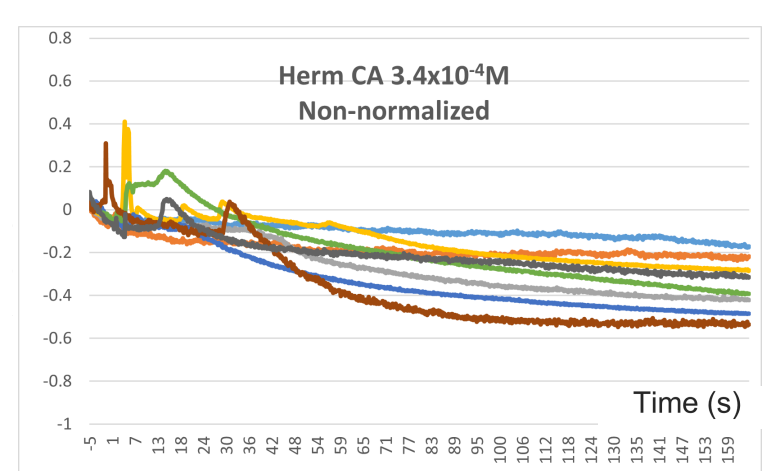

Supplement: Supplementary file 4 — Additional file 4: Figure S4. Cyclohexyl acetate–evoked calcium responses in male AWCon neurons (non-normalized mean ± s.e.m., individual traces, and raw traces). [file 12915_2026_2510_MOESM4_ESM.pdf]

**A****CA-SP**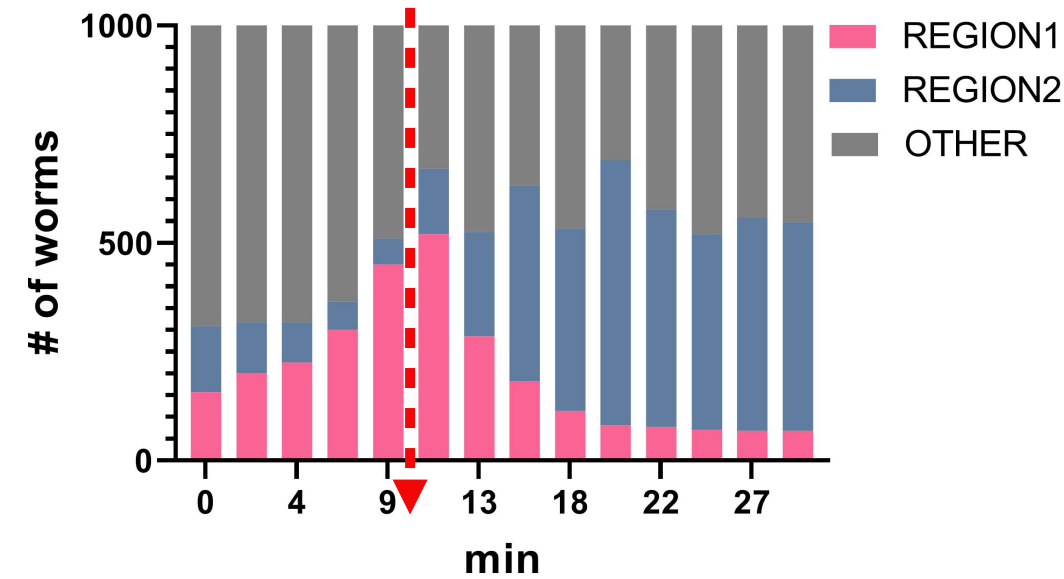**B****VSP-VSP**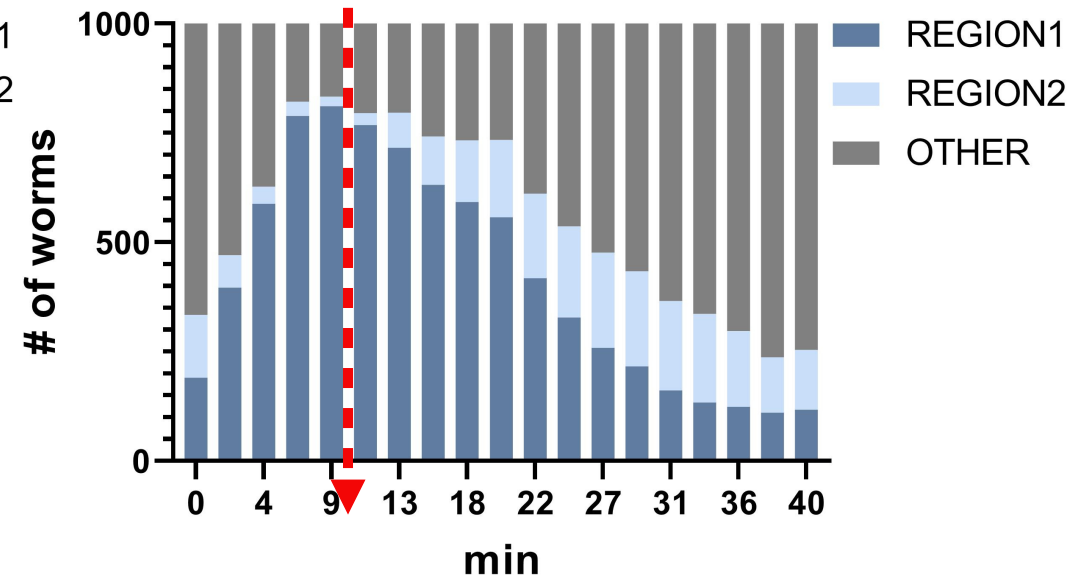**C****DA-DA**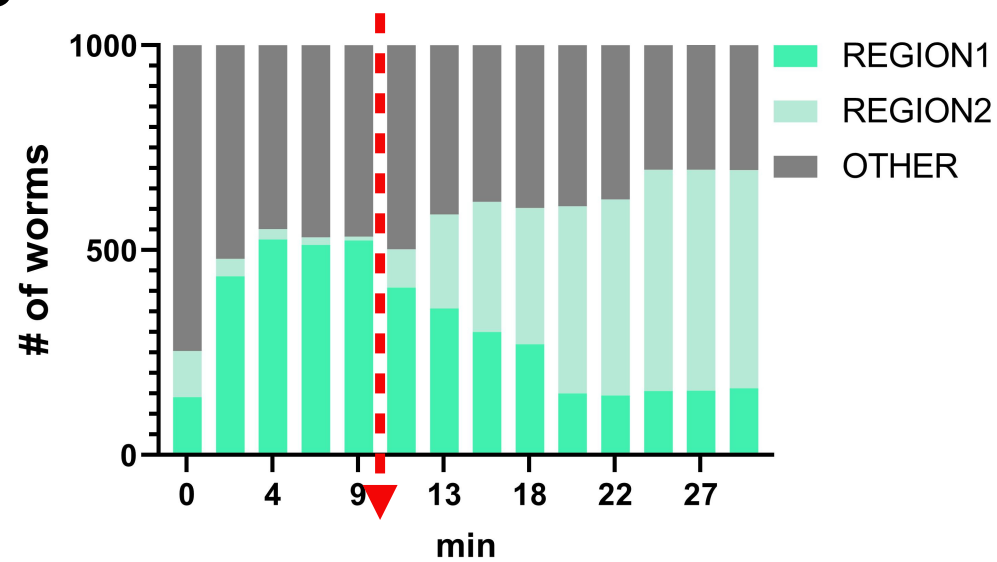

Supplement: Supplementary file 5 — Additional file 5: Figure S5. Pre-exposure/adaptation assays showing attraction after CA pre-exposure, loss of attraction after VSP pre-exposure, and persistence of attraction after diacetyl pre-exposure; timing of chemical switch indicated. [file 12915_2026_2510_MOESM5_ESM.pdf]
